# Gas Forming Pyogenic Liver Abscess Diagnosed by Point of Care Ultrasound

**DOI:** 10.24908/pocus.v9i1.16854

**Published:** 2024-04-22

**Authors:** Wei Ven Chin, Mae Jane Khaw

**Affiliations:** 1 Acute Internal Medicine, Sarawak General Hospital Sarawak Malaysia; 2 Medical Department, Limbang Hospital Sarawak Malaysia

**Keywords:** Case report, Liver abscess, Gas-forming abscess, Point of care ultrasound (POCUS), Bedside ultrasound

## Abstract

Gas-forming pyogenic liver abscess (GFLPA) carries a high mortality rate. Early identification of the source of infection in sepsis results in better survival. Bedside point of care ultrasound (POCUS) can be used to help localize a source of infection. A 59-year-old man presented with systemic inflammatory response syndrome (SIRS) and was diagnosed with GFLPA on the initial encounter via clinical assessment and POCUS examination. After commencing antibiotics, optimal glucose control, adequate fluid resuscitation, and early infective source control, he achieved full recovery and was followed up in outpatient medical and surgical clinics. This case illustrates the role of POCUS as a diagnostic tool in sepsis and raises awareness among clinicians to recognize the features of GFLPA on POCUS.

## Introduction

The Asia Pacific region accounts for 60% of the world's population with 4.6 billion people. National sepsis rates in this region range from 120 to 1,600 per 100,000, and sepsis-related mortality rates of up to 35% 1. The annual incidence rate of pyogenic liver abscess (PLA) ranges from 2 to 45 incidents per 100,000 hospital admissions worldwide 2. PLA is further subdivided into gas-forming pyogenic liver abscess (GFPLA) and non-GFPLA. GFPLA has a greater fatality rate than non-GFPLA, with symptoms ranging from mild fever and abdominal pain to severe sepsis accompanied by a ruptured abscess, that culminates in fulminant peritonitis. 

Point of care ultrasound (POCUS) can be used to localize a source in the evaluation of sepsis, such as PLA. On POCUS examination, liver abscesses are often poorly demarcated and have a variable appearance, ranging from mostly hypoechoic – with or without some internal echoes – to hyperechoic 3. We report a case of GFPLA that was diagnosed by POCUS, resulting in earlier percutaneous drainage for infective source removal. 

**Figure 1:**
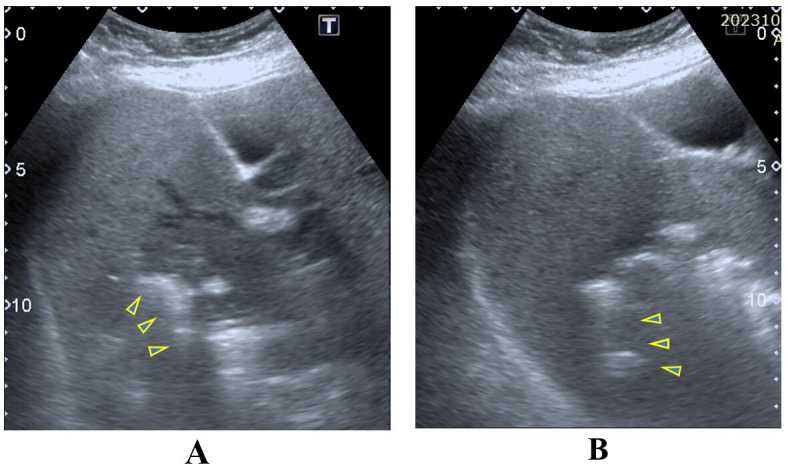
POCUS image A of gas-forming pyogenic liver abscess (GFPLA) demonstrates an ill-defined margin solitary lesion with heterogenous echogenicity. POCUS image B demonstrates brightly echogenic reflectors with posterior reverberation artefacts noted within the lesion. Yellow triangles on both images show the posterior reverberation artefacts.

A 59-year-old man with hypertension presented to the emergency department with fever, chills, and rigors for 5 days, along with lethargy and poor oral intake. He presented to the emergency department with a heart rate of 110bpm, blood pressure of 101/62 mmHg, respiratory rate of 20 breaths/min, oxygen saturation of 98% on room air, and a temperature of 36.5 degrees C. On physical examination, his lungs were clear, there were no murmurs on cardiac auscultation, and his abdomen was soft and non-tender, with no pedal edema. He had dry mucous membranes and concentrated urine. Laboratory examination revealed leukocytosis with a white cell count of 14.2 x 10^3^/μL and thrombocytopenia with a platelet count of 126 x 10^3^/μL. His hemoglobin level was 10.4g/dl, sodium level was 123mmol/L, potassium level was 4.9mmol/L, and his renal profile revealed acute kidney injury with urea of 90mg/dL and creatinine of 2.79mg/dL (normal range 0.67-1.18mg/dL. Liver function tests revealed mildly elevated transaminases with aspartate transaminase (AST) 89u/L and alanine transaminase (ALT) 57u/L, total bilirubin (TB) 0.64mg/dL and direct bilirubin 0.18md/dL, and albumin 27g/L. Blood glucose levels were 252mg/dL, indicating hyperglycemia. Further infective screening revealed a negative leptospirosis serology, a negative dengue NS1 antigen, a negative dengue IgM and IgG, and no malaria parasites on blood film microscopy analysis. POCUS was conducted to look for the source of the infection. Cardiac POCUS revealed no large valvular vegetation, but abdominal POCUS revealed a solitary liver lesion with the features of an ill-defined margin with heterogeneous echogenicity and brightly echogenic reflectors, with posterior reverberation artifacts noted within the lesion (Figure 1). 

**Figure 2:**
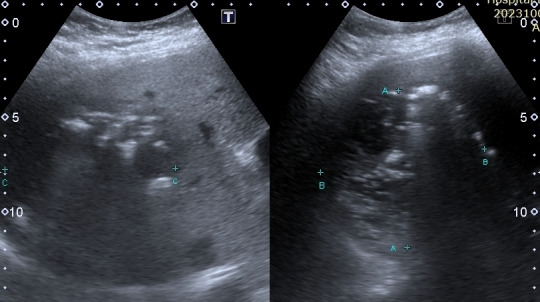
The size of the GFPLA with distance A measuring 8.3cm, distance B measuring 8.7cm, and distance C measuring 9.0cm.

**Figure 3:**
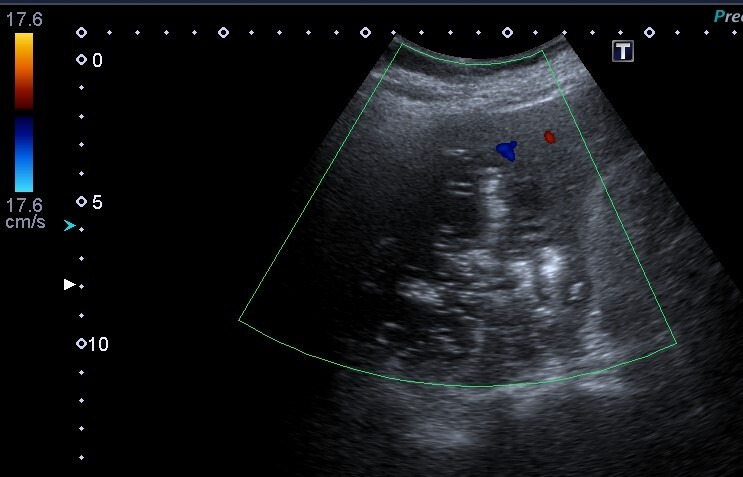
GFPLA with colour doppler, demonstrating no colour doppler signal within indicating the absence of centralperfusion

**Figure 4:**
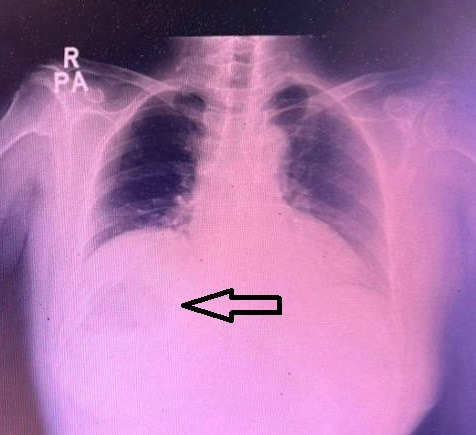
Chest x-ray with gas shadow (depicted by the black arrow) seen beneath the right hemidiaphragm.

The size of the liver lesion measured 8.3cm x 8.7cm x 9.0cm (Figure 2) and the colour doppler demonstrated no colour doppler signal within, indicating the absence of central perfusion (Figure 3). Chest x-ray (CXR) showed gas shadow beneath the right hemidiaphragm (Figure 4). 

**Figure 5:**
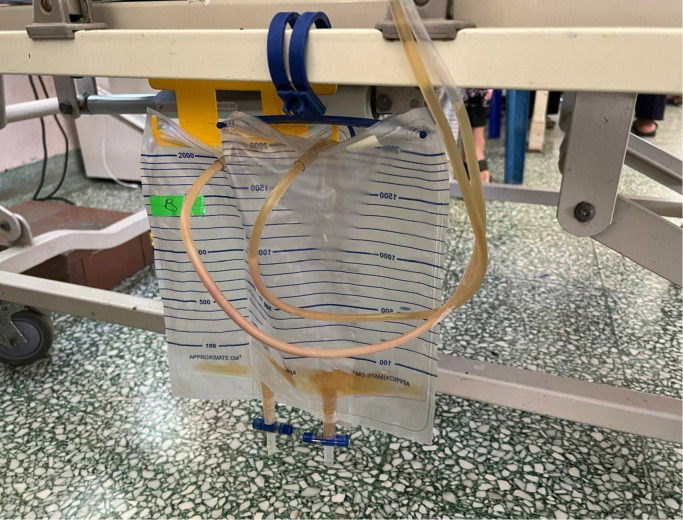
Pus drained from the gas-forming pyogenic liver abscess (GFPLA) via percutaneous drainage catheter.

The presence of relative hypotension, tachycardia, leucocytosis, fever, POCUS features of the liver lesion, and CXR findings favoured the diagnosis of sepsis caused by GFPLA. Standard sepsis management commenced using antibiotics, adequate fluid resuscitation, and optimal glucose control. An urgent referral to radiological and surgical teams was made to expedite drainage for infective source control. Percutaneous drainage catheters were inserted and pus was drained (Figure 5). Both his blood culture and pus culture grew Klebsiella pneumoniae. He subsequently made a full recovery, and was discharged with an oral hypoglycemic agent, an antihypertensive drug, and antibiotics. After being discharged, he was given follow-up appointments at the medical and surgical clinics to optimize his blood pressure, for diabetes management, and for a reassessment of the abscess following a six-week antibiotic course.

GFPLA was described by Smith in 1944 4. The common culprit organisms in pyogenic liver abscess are Escherichia coli and Klebsiella pneumoniae. Diabetes mellitus is an important predisposing factor for pyogenic liver infection 5. High glucose concentration in tissue and compromised immunity in diabetic patients permits proliferation of these microbes. Klebsiella pneumoniae infection in patients with diabetes is a risk factor for the development of GFPLA 6. GFPLA is associated with a high mortality rate and high frequency of septic shock and bacteraemic presentations 7. In their study, Chou et al. describe that the duration of symptoms was shorter, and the incidence of septic shock was higher in the GFLPA group than non-GFLPA group 8. GFLPA generally has a less favourable outcome as it is associated with rapid clinical deterioration and a high mortality rate. Standard sepsis therapy of early initiation of antibiotics, glucose optimization, appropriate fluid management, inotropic supports, nutritional support, and early drainage are crucial in determining the survival of a patient with GFLPA. Thus, early identification of the GFLPA by ultrasound or POCUS examination can be crucial.

According to a recent retrospective study by Lin et al., ultrasonography has a sensitivity of 85.8% for identifying pyogenic liver abscess 9. Utilizing computed tomography imaging in addition to ultrasonography yielded a remarkable 100% diagnosis rate for GFLPA. On ultrasound, liver abscesses are typically poorly demarcated with a variable appearance. This can range from predominantly hypoechoic (with some internal echoes) to hyperechoic, while the colour doppler will demonstrate the absence of central perfusion. GFPLA can appear as a diffuse hyperechoic spot with acoustic shadowing, or as a hyperechoic lesion with reverberation. 

POCUS played a pivotal role in this patient’s care, aiding in a rapid diagnosis. Early identification of liver abnormality on POCUS resulted in an expedited diagnosis and management, which likely contributed to a favorable outcome.

## Statement of ethics approval/consent

Informed consent and permission for publication of the case and ultrasound images was obtained from the patient.
